# Estimating the Continuous-Time Dynamics of Energy and Fat Metabolism in Mice

**DOI:** 10.1371/journal.pcbi.1000511

**Published:** 2009-09-18

**Authors:** Juen Guo, Kevin D. Hall

**Affiliations:** Laboratory of Biological Modeling, National Institute of Diabetes and Digestive and Kidney Diseases, Bethesda, Maryland, United States of America; ETH Zurich, Switzerland

## Abstract

The mouse has become the most popular organism for investigating molecular mechanisms of body weight regulation. But understanding the physiological context by which a molecule exerts its effect on body weight requires knowledge of energy intake, energy expenditure, and fuel selection. Furthermore, measurements of these variables made at an isolated time point cannot explain why body weight has its present value since body weight is determined by the past history of energy and macronutrient imbalance. While food intake and body weight changes can be frequently measured over several weeks (the relevant time scale for mice), correspondingly frequent measurements of energy expenditure and fuel selection are not currently feasible. To address this issue, we developed a mathematical method based on the law of energy conservation that uses the measured time course of body weight and food intake to estimate the underlying continuous-time dynamics of energy output and net fat oxidation. We applied our methodology to male C57BL/6 mice consuming various *ad libitum* diets during weight gain and loss over several weeks and present the first continuous-time estimates of energy output and net fat oxidation rates underlying the observed body composition changes. We show that transient energy and fat imbalances in the first several days following a diet switch can account for a significant fraction of the total body weight change. We also discovered a time-invariant curve relating body fat and fat-free masses in male C57BL/6 mice, and the shape of this curve determines how diet, fuel selection, and body composition are interrelated.

## Author Summary

The unrelenting obesity epidemic has resulted in intensive basic scientific investigation into the molecular mechanisms of body weight regulation—with the mouse being the organism of choice for such studies. We know that any mechanism of body weight regulation must exert its effect by influencing food intake, energy output, fuel selection, or some combination of these factors over extended time scales (∼weeks for mice). While food intake and body weight can be frequently measured in mice, current methods prohibit corresponding measurements of energy output or fuel selection on such long time scales. We address this deficiency by developing a mathematical method that quantitatively relates measurements of food intake, body weight and body fat to calculate the dynamic changes of energy output and net fat oxidation rates during the development of obesity and weight loss in male C57BL/6 mice. The mathematical model is based on the law of energy conservation, makes very few assumptions, and provides the first continuous-time estimates of energy output and fuel selection over periods lasting many weeks. Application of our methodology to various mouse models of obesity will improve our understanding of body weight regulation by placing molecular mechanisms in their whole-body physiological context.

## Introduction

Mouse models of obesity have become critically important research tools for discovering molecular mechanisms of body weight regulation. But understanding these mechanisms in the context of whole-body physiology requires knowledge of food intake, energy output, and fuel selection [1]. Furthermore, measurements made at an isolated time point cannot explain why body weight has its present value since body weight is determined by the past history of energy and macronutrient imbalance [2]. While food intake and body weight changes can be measured frequently over several weeks (the relevant time scale for mice), correspondingly frequent measurements of energy output and fuel selection are not currently feasible.

Expensive indirect calorimetry systems can be used to measure energy expenditure and respiratory exchange over periods of a few days and most systems require removing mice from their normal environment which can alter their behavior [3]. Alternatively, the doubly labeled water method can give an estimate of average energy expenditure, but this method requires specialized equipment for sample analysis as well as prior knowledge of fuel selection as measured by the respiratory quotient (RQ) [4]. Furthermore, significant quantities of blood need to be collected which could impact the behavior of the mouse and makes repeat measurements untenable [4].

Here, we present a mathematical method that quantitatively relates food intake, body weight and body fat to calculate the dynamic changes of energy output and net fat oxidation rates during the development of obesity and weight loss in male C57BL/6 mice. The mathematical model is based on the law of energy conservation, makes very few assumptions, and provides the first continuous-time estimates of energy output and fuel selection over periods lasting many weeks. Our methodology also revealed the relationship between diet, fuel selection, and body composition change in male C57BL/6 mice by identifying a time-invariant curve relating body fat and fat-free masses.

## Results

### Body Composition and the Relationship between Body Fat and Fat-Free Mass

**Figure 1:**
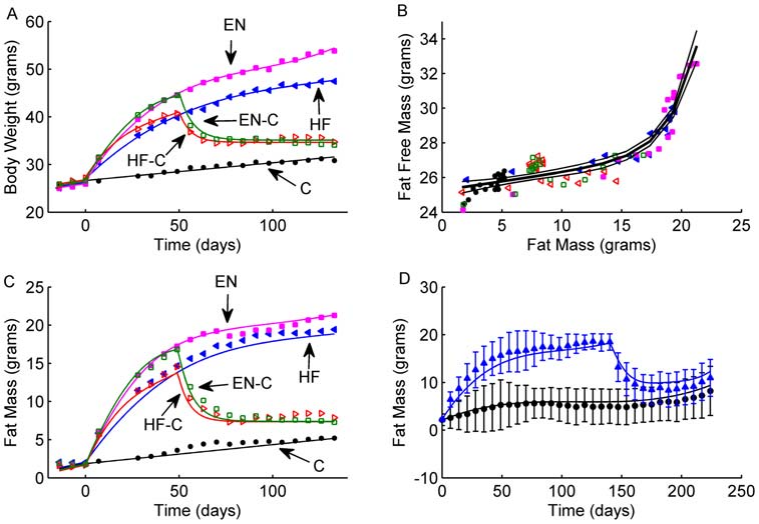
C57BL/6 mouse data and model predictions for fat mass. Panel A: measured body weight in male C57BL/6 mice from 5 diet groups: the C group on chow diet (closed black circles), the HF group on high fat diet (closed blue triangles), the EN group on high fat diet plus Ensure (closed pink squares), the HF-C group on high fat diet for 7 wk followed by a switch to chow (open red triangles), and the EN-C group on high fat diet plus Ensure for 7 wk followed by a switch to chow (open green squares). The solid lines labeled are fitted curves to body weight data. Panel B: Measured body fat mass (F) and adjusted fat free mass (FFM) from the 5 groups designated using the same markers as in Panel A. These data were fit to a single exponential function for all groups: 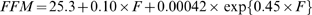 (thick line with thin lines representing 95% confidence intervals). Panel C: measured fat mass from the 5 groups where the data point markers was the same as in Panel A. The solid lines are model predictions for fat mass. Panel D: measured fat mass from a separate experiment with two groups of male C57BL/6 mice: group one on chow diet for 33 wk (closed black circles); group two on high fat diet for 20 wk followed by a switch to chow (closed blue triangles). The error bars represent 95% confidence interval for the measurements. The curves represent the model predictions for fat mass in groups one (black) and two (blue).

As previously described [5], male C57BL/6 mice were given *ad libitum* access to standard chow (C), high fat diet (HF), or high fat diet plus liquid Ensure (EN) for 19 weeks, while some mice were fed the high fat or the high fat plus Ensure for 7 weeks before being switched back to chow for the remaining 12 weeks (HF-C and EN-C, respectively). Figure 1A shows the body weight changes of the various groups during the development of obesity on the HF and EN diets as well as the weight loss and persistent obesity of the HF-C and EN-C groups following a switch back to the chow diet at 7 weeks (error bars have been omitted for clarity). A single curve was able to describe the adjusted fat-free mass as a function of body fat mass for all groups at all time points (Figure 1B) and is analogous to the curve discovered by Forbes describing human body composition change [6]. Our mathematical model used this fitted curve along with the body weight data to compute the body fat mass changes (Figure 1C). Without adjusting any parameters, the model also accurately predicted the fat mass changes measured in a separate experiment with high-fat feeding of C57BL/6 mice followed by a switch to chow after 20 weeks (Figure 1D).

### Continuous-Time Estimates of Energy Output

**Figure 2:**
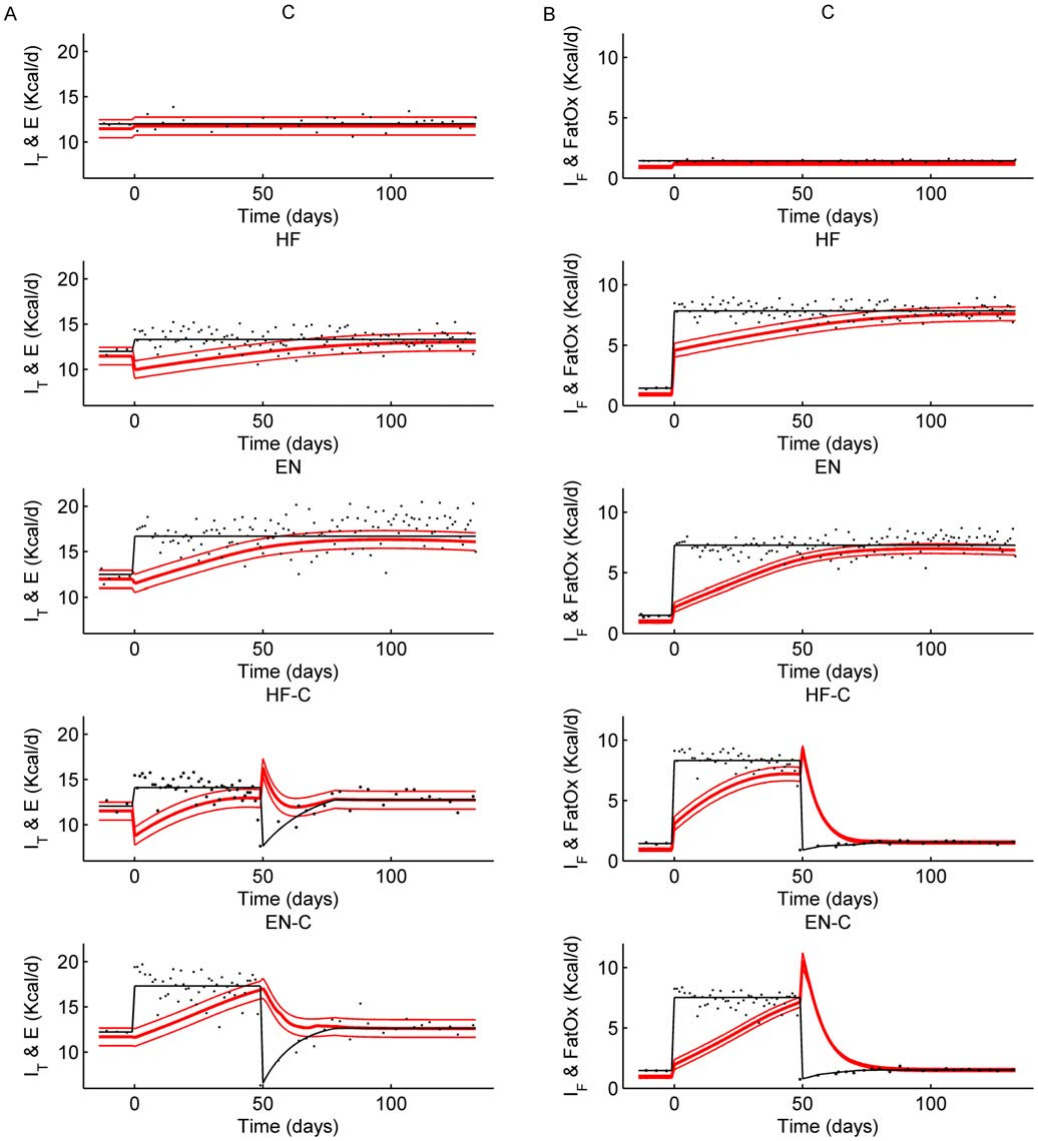
Total energy intake (I_T_), fat intake (I_F_), and model predictions for energy output (E) and net fat oxidation rates (FatOx) in male C57BL/6 mice. Each row represents one of the five diet groups (C, HF, EN, HF-C, EN-C). Panel A: energy intake measurements (black data points) were fit according to the thin black curves and used as inputs to our model. The model predictions for energy output are shown as thick red curves along with 95% confidence intervals (thin red curves). Panel B: measured fat intake (black data points), average fat intake over each diet period (thin black curves), and model predictions for net fat oxidation rate (red curves) along with 95% confidence intervals.

Our model calculated the first continuous-time estimates of the energy output dynamics underlying the observed body weight changes (Figure 2A). The 95% confidence interval surrounding the calculated energy output rates resulted primarily from variability of the measured energy intake rate (individual data points are depicted along with the average black curve used for each group) but also included the effect of body composition variability (Figure 1B). The HF and HF-C groups had a transient decrease of energy output at the onset of high fat feeding at 0 days. In contrast, the EN and EN-C groups did not show a significant transient reduction of energy output at the onset of the high energy diet. Energy output gradually increased with weight gain in all of the groups. Following the return to the chow diet, the HF-C group had a transient increase of energy output which was not seen in the EN-C group. Note that these transient changes account for significant fractions of the overall energy imbalances and would be difficult to detect using indirect calorimetry or doubly labeled water methods.

### Continuous-Time Estimates of Fuel Selection

Net fat oxidation rates increased sharply at the onset of high fat feeding in the HF and HF-C groups, but did not rise sufficiently to match the increase of fat intake (Figure 2B). Interestingly, despite similar increases of fat intake in the EN and EN-C groups compared with the HF and HF-C groups, the initial increase of net fat oxidation was significantly attenuated. Net fat oxidation gradually increased in all the groups as body weight increased. Following the switch to chow, there was a transient increase of net fat oxidation in both HF-C and EN-C groups before falling to match the low level of fat intake after a few weeks.

**Figure 3:**
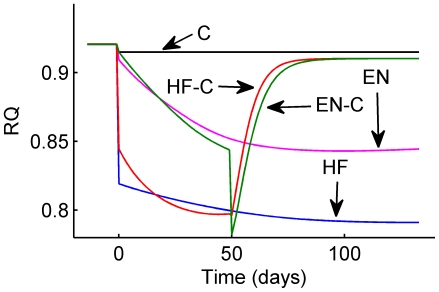
Model predictions for the daily respiratory quotient (RQ). The C group was fed a chow diet (black), the HF group was fed a high fat diet (blue), the EN group was fed a high fat diet plus Ensure (pink), the HF-C group was fed a high fat diet for 7 wk followed by a switch to chow (red), and the EN-C group was fed a high fat diet plus Ensure for 7 wk followed by a switch to chow (green).

A useful measure of fuel selection is the respiratory quotient, RQ, where a value of 0.7 reflects a state of pure fat oxidation whereas a value of 1.0 reflects a state of pure carbohydrate oxidation and intermediate values represent a fuel selection mixture (see Methods). The estimated 24 hour RQ (Figure 3) demonstrates the impact of both diet and body composition on fuel selection. The HF group had an immediate decrease of RQ due to the diet followed by a slow progressive decrease as body fat gradually increased. The EN group showed little initial change of RQ which then progressively decreased to an intermediate value. After switching to the chow diet, the HF-C group had a rapid increase of RQ towards that of the C group whereas the EN-C group had a transient decrease of RQ before increasing towards the C group.

## Discussion

The mouse has become the most popular organism for investigating molecular mechanisms of body weight regulation. But understanding the physiological context by which a molecule exerts its effect on body weight requires knowledge of energy intake, energy expenditure, and fuel selection. Our simple mathematical method calculates the dynamics of energy output and fuel selection over extended time periods using longitudinal measurements of body weight, food intake, and body composition. We showed that our method can detect both transient changes of energy expenditure and net fat oxidation rates as well as longer timescale changes found with weight gain and loss. Similar methodology has been previously developed by our group to relate human body-composition changes with dynamic adaptations of fuel selection in both adults [7] and infants [8]. The method is especially well-suited for mouse studies because food intake can be accurately measured over the extended time periods required to measure significant changes of body weight and body fat. While we have applied the model to data averaged within groups of mice, it would be also interesting to examine individual mouse trajectories as a way of investigating inter-individual variability.

Our equations extract information about energy output that is already present in the body weight and food intake data. Other than the law of energy conservation, the only assumption was that the relationship between changes of body fat and fat-free mass were described by a well-defined function in accordance with the Forbes theory of body composition change [6]. This assumption was confirmed in the present study for mature male C57BL/6 mice (Figure 1B) and we hypothesize that genetic manipulations can alter the shape of this function. However, once the function has been determined we showed that it provided accurate estimates of body fat changes in an independent feeding experiment using body weight measurements alone (Figure 1D). Therefore, knowledge of the Forbes curve for a given mouse model eliminates the need for frequent body composition measurements.

To estimate the net fat oxidation rate and RQ, an additional assumption regarding carbohydrate balance was required (see Methods). We found that the Forbes function (Figure 1B) determined the relationship between food intake, body composition change, and net fat oxidation rate [7]. While both humans and mice have Forbes functions that increase with body fat mass, the concavity of the curves is opposite [6]. Therefore, great caution should be exercised when extrapolating fuel selection results in mice to predict human responses. The physiological reason for this difference is presently unclear. Our research group is actively engaged in developing detailed models of the complex interactions between carbohydrate, fat, and protein metabolism in humans [9] to better understand the relationship between the physiological drivers of fuel selection and the Forbes body composition curve. We plan to develop similar models in mice to help understand these relationships and the differences between the species.

In contrast to our method, currently available techniques for estimating energy expenditure are expensive, involve a plethora of assumptions, and can impact the behavior of the mice [3],[4]. These factors make it common to find reports of energy expenditure rates that are quantitatively inconsistent with the measured energy intake and body weight changes found in mice that were not subjected to these procedures. As an illustrative example, consider the recent publication by Funato et al. where the energy intake rate of the wild type mice was at least 17 kcal/d and the energy expenditure measured by indirect calorimetry was less than 5 kcal/hr/(kg BW)^0.75^. This translates to an absolute expenditure rate of less than 10.7 kcal/d for a mouse that was at most 40 grams at the time of measurement [10]. Such a large positive energy balance would translate to a rate of weight change of at least 4.7 g/week (if all excess energy was deposited as fat) versus the measured weight gain which was less than 1 g/week. The purpose of this example is not to criticize the work of Funato et al., but rather to highlight how even careful indirect calorimetry and food intake measurements can lead to estimates of energy imbalance that are inconsistent with the weight gain measurements.

Our own attempt to use indirect calorimetry to validate the model predictions of energy expenditure and fuel selection highlighted two important issues. First, the mice that were consuming the high energy diets lost significant amounts of weight when moved to the indirect calorimetry cages indicating that their behavior was not representative of the mice not subjected to the procedure. Second, the measured energy expenditure rates were unrealistically high compared to the model predictions for all groups of mice. In fact, the measured energy expenditure rate was higher than the measured energy intake in the chow-fed mice that did not lose weight (an impossibility) and greatly exceeded the expenditure required to explain the weight loss in the mice fed the high energy diets. These discrepancies led us to diagnose a technical problem with the indirect calorimetry equipment. Thus, we were unable to validate the model estimates of energy expenditure and fuel selection.

The field of farm animal nutrition has a long and rich history of using mathematical modeling to analyze animal growth and identify nutritional factors that potentially limit growth rate [11]–[14]. The simplest models describe the efficiencies of various diets in their ability to deposit body energy, often specified in terms of body fat and protein [11],[13],[14]. Inputs to such models include energy intake, body weight, and the rates of body fat and protein deposition. The model outputs include the efficiencies of protein and fat deposition as well as the so-called maintenance energy requirement which is roughly defined as the energy intake required when the animal is not growing. An alternative representation uses energy intake, body weight, total energy expenditure (by calorimetry methods), and protein deposition rate (via nitrogen balance) as model inputs and predicts the maintenance energy requirement, fat deposition rate, and body protein and fat deposition efficiencies.

At the next level of complexity, animal growth models prescribe an energy partitioning rule that specifies how body protein will accumulate for a given food intake rate as a function of body weight, age, or body protein. Energy partitioning rules are often complex [12],[13], but can be thought of as similar to the Forbes function that specifies how energy imbalances are partitioned between body fat and fat-free mass. A significant difference is that our approach is applied to mature mice whose overall growth rate was minimal despite their ability to gain and lose fat-free mass in response to the various diets.

Once the partitioning rule is specified, the outputs of animal growth models include body fat mass, maintenance energy requirement, as well as body fat and protein deposition efficiencies given the food intake and body weight as model inputs. In contrast, our model outputs are body fat mass, fuel selection, and total energy expenditure which are more relevant for mouse obesity studies and avoids the known problem of arbitrarily distributing total energy expenditure between tissue deposition costs versus maintenance energy requirements [11], [14]–[16]. Animal growth models have often used power-law functions of body weight to model the maintenance energy requirements that were previously calculated using the above methods. Once specified, the model of maintenance energy requirements can be used along with the calculated efficiencies of protein and fat deposition and the energy partitioning rule to predict body weight and body fat change as a function of the food intake [11],[14]. We are presently developing a model of total energy expenditure in mice that will allow prediction of body weight and composition changes as well as fuel selection when food intake is the only input to the model.

A weakness of our methodology is that it does not distinguish the various components of energy output including resting metabolic rate, thermic effect of feeding, adaptive thermogenesis, physical activity, or any changes of energy excreted in urine and feces that are unaccounted for by the estimates of diet metabolizability. Furthermore, the method does not operate on a within-day time scale and therefore cannot address changes between day versus night or transitions between fed and fasted states. Indirect calorimetry is required to address these issues and would provide important information for the interpretation of our calculated longer-term estimates of energy output and fuel selection. We believe that the combination of our continuous-time methodology with indirect calorimetry measurements at judiciously chosen time points can be applied to various mouse models of obesity as a powerful tool for characterizing the metabolic dynamics underlying experimentally observed body weight changes.

## Methods

### Ethics Statement

We certify that all applicable institutional and governmental regulations concerning the ethical use of animals were followed during this research. All procedures were approved by the National Institute of Diabetes and Digestive and Kidney Diseases Animal Care and Use Committee.

### Mouse Experiment

Full details of the experiment were previously described [5]. Briefly, forty seven 3 month old male C57BL/6 mice weighing 25.9±1.2 g (The Jackson Laboratory, Maine) were housed individually and randomly assigned to five weight-matched groups: 1) C group (N = 12) continued on the chow diet; 2) HF group (N = 12) on a high fat diet (F3282; Bio-Serv Inc., NJ; 5.45 kcal/g with 14% energy derived from protein, 59% from fat, and 27% from carbohydrate); 3) EN group (N = 11) on the high fat diet plus liquid Ensure (Abbott Laboratories, Kent, UK), which had an energy density of 1.06 kcal/ml with 14% of energy derived from protein, 22% from fat, and 64% from carbohydrate; 4) HF-C group (N = 6) switched from high fat to chow after 7 weeks; 5) EN-C group (N = 6) switched from high fat plus Ensure to chow after 7 weeks. All animals received free access to water and food throughout the study. The high fat diet was provided using Rodent CAFÉ_TM_ feeders (OYC International, Inc., MA), and liquid Ensure was provided in a 30-ml bottle with a rodent sip tube (Unifab Co., MI) and liquid intake was measured every day. Solid food intake was corrected for any visible spillage and was measured every day for the high fat diet and every other day for the chow diet using a balance with a precision of 0.01 g (Ohaus model SP402). Body composition was measured once per week using 1H NMR spectroscopy (EchoMRI 3-in-1, Echo Medical Systems LTD, Houston, TX) after body weight was determined.

### Mathematical Model

We begin with the law of energy conservation, also known as the energy balance equation:
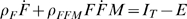
(1)where *F* is the body fat mass, *FFM* is the fat-free mass defined as the measured body weight, *W*, minus the fat mass, and 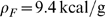 and 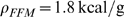 are the energy densities for changes in fat and fat-free masses, respectively [17]. *I_T_* is the total metabolizable energy intake rate corrected for spillage, and *E* is the energy output rate. We distinguish the energy output rate from the energy expenditure rate since we did not measure any changes of energy excreted in urine or feces. In other words, if the metabolizable energy content of each diet is constant then our calculation of the energy output is equivalent to energy expenditure.

Analogous to the Forbes theory of human body composition change [6], we hypothesized that there is a well-defined, time-invariant function, *α*, that describes the relationship between changes of *FFM* and *F* in male C57BL/6 mice:
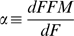
(2)Once the function *α* is specified, equation (1) can be solved for the energy output rate as a function of the measured energy intake rate and the rate of body weight change as follows:
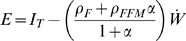
(3)The fat mass is given by solving the following differential equation:
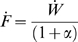
(4)Alternatively, if the Forbes assumption does not apply for a given mouse model (for example, during periods of significant growth), a curve could be directly fit to the measured fat mass time series data and used in place of equation 4. While this procedure would give equivalent results, it necessitates frequent body composition measurements for every experiment.

Note that very few assumptions were made in the development of our equations to estimate energy output. All of the above equations were derived from the law of energy conservation (1) and the only assumption was that there exits a well-defined Forbes relationship, α, relating changes of body fat and fat-free masses – an assumption that was directly confirmed by comparison to measured body composition data.

Since we are also interested in fuel selection, we must consider the fates of dietary macronutrients including their oxidation rates, storage in the body, as well as major inter-conversion fluxes where carbohydrate can be converted to fat (i.e., de novo lipogenesis) and amino acids can be converted to the carbohydrate glucose (i.e., gluconeogenesis). The following macronutrient balance equations represent these changes:
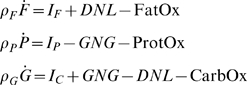
(5)where *P* is body protein, *G* is glycogen, *GNG* is the gluconeogenic rate, *DNL* is the de novo lipogenic rate, and *I_F_*, *I_P_* and *I_C_* are the intake rates of dietary fat, protein and carbohydrate, respectively. The oxidation rates of fat, protein, and carbohydrate (FatOx, ProtOx, and CarbOx, respectively) sum to the total energy output, *E*.

To simplify the macronutrient balance equations, we note that glycogen stores are small, especially when compared with daily carbohydrate intake rates. For example, humans have a glycogen pool size of about 500 g which is equivalent to the typical amount of carbohydrate consumed over ∼2 days and equilibrates on a time scale of ∼1 day [9],[18]. The equilibration time is likely even more rapid in mice since they typically consume carbohydrate at a rate of ∼2 g/d and their glycogen stores are probably less than 0.6 g (assuming maximal glycogen pool sizes of 8% of liver weight and 0.6% of muscle weight as observed in rats [19] and assuming that mouse liver is less than 5 g and muscle is less than 30 g [5]). Thus, over the time-scale of interest the system is in a state of average carbohydrate balance:
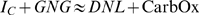
(6)Therefore,
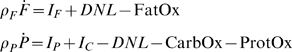
(7)If we define the net fat oxidation rate as follows:
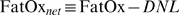
(8)then the equation for body protein change becomes:
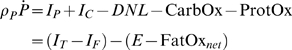
(9)Finally, we assume that *FFM* is proportional to body protein such that
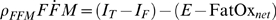
(10)Therefore, we have a simple a two-compartment macronutrient partitioning model which we have previously shown has an invariant manifold as its attractor [20]:
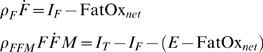
(11)From equations 4 and 11, the net fat oxidation rate can be written as a function of the measured fat intake rate and the rate of body weight change:
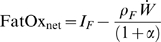
(12)Note that the carbohydrate balance assumption was only required to calculate the estimate of net fat oxidation, but was not required to calculate the energy output rate.

The shape of the Forbes curve has direct implications for how fat oxidation rate is related to changes of body fat. This can be seen by calculating the partial derivative of the net fat oxidation rate with respect to body fat:
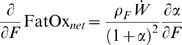
(13)Interestingly, this quantity has opposite sign in humans versus mice. Thus, great care must be taken when fuel selection measurements in mice are extrapolated to humans.

The respiratory quotient, *RQ*, is the carbon dioxide production rate divided by the oxygen consumption rate and was approximated by:
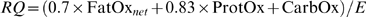
(14)This approximation assumes a negligible contribution of de novo lipogenesis and gluconeogenesis which is reasonable since these fluxes act to offset each other with respect to CO_2_ production. Since the carbohydrate oxidation rate is approximately equal to the carbohydrate intake rate on long time scales, the calculated *RQ* may have slight inaccuracies during rapid transitions immediately after diet switches, but will be reasonably accurate thereafter.

To apply our mathematical model to data from our mouse experiment, food intake measurements were averaged over each diet period and we assumed stepwise transitions immediately after each diet switch followed by a smooth approach to the average intake of the final diet period. These curves are depicted as solid black lines in Figure 2 and represent the average of the individual intakes shown by the data points. Body weight measurements for the C, HF, and EN groups of mice were fit using third order polynomial functions of time, as depicted by the solid curves in Figure 1A. Following the diet switch in the HF-C and EN-C groups, the body weight curves were fit to exponential functions. The rates of change of body weight were then calculated by computing derivatives of the fitted curves. Other than their ability to adequately describe the model input data, the precise mathematical form of these curves is not important.

The Forbes body composition function, *α*, was fit to an exponential function of the body fat mass as shown in Figure 1B. Specifically, we assumed that the individual data points for fat-free mass versus body fat for each group of mice were described by the following equation:
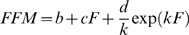
(15)The Forbes function, *α*, is then given by:
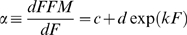
(16)Since the intercept parameter, *b*, does not influence the Forbes function, we adjusted the *FFM* data for each group by subtracting the difference between the calculated intercept parameter for each group and its average value across groups. We then simultaneously fit the adjusted *FFM* data from all groups to arrive at our final Forbes function used for all of the groups.

The parameter values for the Forbes body composition function 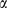 were determined via a Markov Chain Monte Carlo (MCMC) method [21] implemented in MATLAB (version R2008a; MathWorks Inc, Natick, MA). To approximate the posterior distribution of the parameters in the Forbes 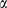 function (equation 16), we drew 100,000 MCMC samples of parameter values, of which the first 30000 were discarded as burn-in period; afterwards one fifth of the rounds were retained. Parameter sets were drawn from a proposal density that were normally distributed and centered on the previous value. The variance of the proposal density was tuned for an average acceptance rate of ∼0.25 during the burn-in period. The convergence of the chain was assessed both by visual inspection of the trace plots for all the parameters and through the Geweke test [22]. At each sampling, the probability of accepting the new parameter set given current parameter set was 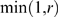 where r is the Metropolis ratio [21]. The posterior distribution of energy output (equation 3) was calculated from the joint distribution of the parameters in the 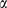 function and the energy intake in each group of the animals assuming no correlation existed between the two distributions. The energy intake in each group of animals was normally distributed with a standard error of 0.39, 0.39, 0.41, 0.55, and 0.55 Kcal/d for the C, HF, EN, F-C, and EN-C groups, respectively. The 95% confidence intervals of the predicted energy output were obtained by calculating the 2.5^th^ and 97.5^th^ percentiles of the posterior distribution of energy output.
